# Combined proteomics and single cell RNA-sequencing analysis to identify biomarkers of disease diagnosis and disease exacerbation for systemic lupus erythematosus

**DOI:** 10.3389/fimmu.2022.969509

**Published:** 2022-11-29

**Authors:** Yixi Li, Chiyu Ma, Shengyou Liao, Suwen Qi, Shuhui Meng, Wanxia Cai, Weier Dai, Rui Cao, Xiangnan Dong, Bernhard K. Krämer, Chen Yun, Berthold Hocher, Xiaoping Hong, Dongzhou Liu, Donge Tang, Jingquan He, Lianghong Yin, Yong Dai

**Affiliations:** ^1^ Institute of Nephrology and Blood Purification, the First Affiliated Hospital of Jinan University, Jinan University, Guangzhou, China; ^2^ Clinical Medical Research Center, The Second Clinical Medical College of Jinan University, Shenzhen People’s Hospital, Jinan University, Shenzhen, China; ^3^ College of Natural Science, University of Texas at Austin, Austin, TX, United States; ^4^ Fifth Department of Medicine, University Medical Centre Mannheim, University of Heidelberg, Heidelberg, Germany; ^5^ Department of Nephrology, Charité-Universitätsmedizin Berlin, Berlin, Germany; ^6^ Key Laboratory of Study and Discovery of Small Targeted Molecules of Hunan Province, School of Medicine, Hunan Normal University, Changsha, China; ^7^ Reproductive and Genetic Hospital of China International Trust and Investment Corporation (CITIC)-Xiangya, Changsha, China; ^8^ Institute of Medical Diagnostics (IMD), Berlin, Germany; ^9^ Department of Rheumatology and Immunology, The Second Clinical Medical College of Jinan University, Shenzhen People’s Hospital, Jinan University, Shenzhen, China; ^10^ Guangzhou Enttxs Medical Products Co., Ltd, Guangzhou, Guangzhou, China

**Keywords:** machine learning, biomarker, immune cell, disease exacerbation, disease diagnosis

## Abstract

**Introduction:**

Systemic lupus erythematosus (SLE) is a chronic autoimmune disease for which there is no cure. Effective diagnosis and precise assessment of disease exacerbation remains a major challenge.

**Methods:**

We performed peripheral blood mononuclear cell (PBMC) proteomics of a discovery cohort, including patients with active SLE and inactive SLE, patients with rheumatoid arthritis (RA), and healthy controls (HC). Then, we performed a machine learning pipeline to identify biomarker combinations. The biomarker combinations were further validated using enzyme-linked immunosorbent assays (ELISAs) in another cohort. Single-cell RNA sequencing (scRNA-seq) data from active SLE, inactive SLE, and HC PBMC samples further elucidated the potential immune cellular sources of each of these PBMC biomarkers.

**Results:**

Screening of the PBMC proteome identified 1023, 168, and 124 proteins that were significantly different between SLE vs. HC, SLE vs. RA, and active SLE vs. inactive SLE, respectively. The machine learning pipeline identified two biomarker combinations that accurately distinguished patients with SLE from controls and discriminated between active and inactive SLE. The validated results of ELISAs for two biomarker combinations were in line with the discovery cohort results. Among them, the six-protein combination (IFIT3, MX1, TOMM40, STAT1, STAT2, and OAS3) exhibited good performance for SLE disease diagnosis, with AUC of 0.723 and 0.815 for distinguishing SLE from HC and RA, respectively. Nine-protein combination (PHACTR2, GOT2, L-selectin, CMC4, MAP2K1, CMPK2, ECPAS, SRA1, and STAT2) showed a robust performance in assessing disease exacerbation (AUC=0.990). Further, the potential immune cellular sources of nine PBMC biomarkers, which had the consistent changes with the proteomics data, were elucidated by PBMC scRNAseq.

**Discussion:**

Unbiased proteomic quantification and experimental validation of PBMC samples from two cohorts of patients with SLE were identified as biomarker combinations for diagnosis and activity monitoring. Furthermore, the immune cell subtype origin of the biomarkers in the transcript expression level was determined using PBMC scRNAseq. These findings present valuable PBMC biomarkers associated with SLE and may reveal potential therapeutic targets.

## Introduction

Systemic lupus erythematosus (SLE) is an incurable, remitting, and relapsing systemic autoimmune disease in young women (1). The disease manifestations of SLE are unpredictable, ranging from mild symptoms, such as rash and arthritis, to severe multi-organ involvement (2). This clinical heterogeneity increases the difficulty of disease diagnosis, clinical remission, and personalized treatment. Thus, of importance for improving clinical management is to discover novel molecular biomarkers, beyond autoantibodies and complement proteins, for disease diagnosis and disease exacerbation assessment of SLE.

To date, the established SLE pathophysiological pathway-based approaches for biomarker detection, although useful, are typically biased, largely because of limited screening of novel biomarkers and their associated pathways (3). Blood or urine liquid biopsies are far less invasive and cost-effective procedures that can be scheduled more frequently for disease diagnosis and monitoring. Human serum and urine have been extensively used in many biomonitoring studies to assess SLE biomarkers (4, 5). Peripheral blood mononuclear cells (PBMCs), as noninvasive biological matrices, are not only crucial for abnormal changes in immune cell subsets, but are also central to the pathogenesis of SLE (6–8). PBMCs are thought to have good potential for biomarker detection.

More recently, unbiased approaches have been used for biomarker discovery, including the protein microarray platform (9), which is confined to detecting fewer proteins, and mass spectrometry (10) with limited detection of low-abundance proteins. In contrast to previous methods, a hybrid trapped ion mobility spectrometry (TIMS) quadrupole time-of-flight (Q-TOF) mass spectrometer (MS) with the parallel accumulation-serial fragmentation (PASEF) technique provides a more powerful performance. Briefly, the technique, named four-dimensional label-free quantitative (4D-LFQ), assembles four-dimensional patterns including mass-to-charge ratio (m/z), retention time, ion mobility, and intensity (11). This greatly improves the speed, sensitivity, and flux of proteomic detection and can be applied to screen biomarker combinations. Moreover, single-cell RNA sequencing (scRNA-seq) of PBMCs has the potential to be a robust and unbiased method for profiling the makeup-and cell type–specific transcriptional states of peripheral immune cells at the same time.

This study aimed to identify biomarkers for disease diagnosis and assessment of disease exacerbation in patients with SLE. Using 4D-LFQ technology, a discovery cohort of patients with SLE was investigated for quantitative proteomics in PBMCs. To this end, we developed a machine learning pipeline based on PBMC proteomics data and identified two candidate biomarker combinations for disease diagnosis and disease exacerbation assessment. Another cohort was used to validate these two biomarker combinations *via* enzyme-linked immunosorbent assays (ELISAs). Finally, we identified the expression of each of these PBMC biomarkers in different immune cell types using scRNAseq data from SLE patients and healthy donor PBMC samples.

## Materials and methods

### Patients, sample collection

**Figure 1:**
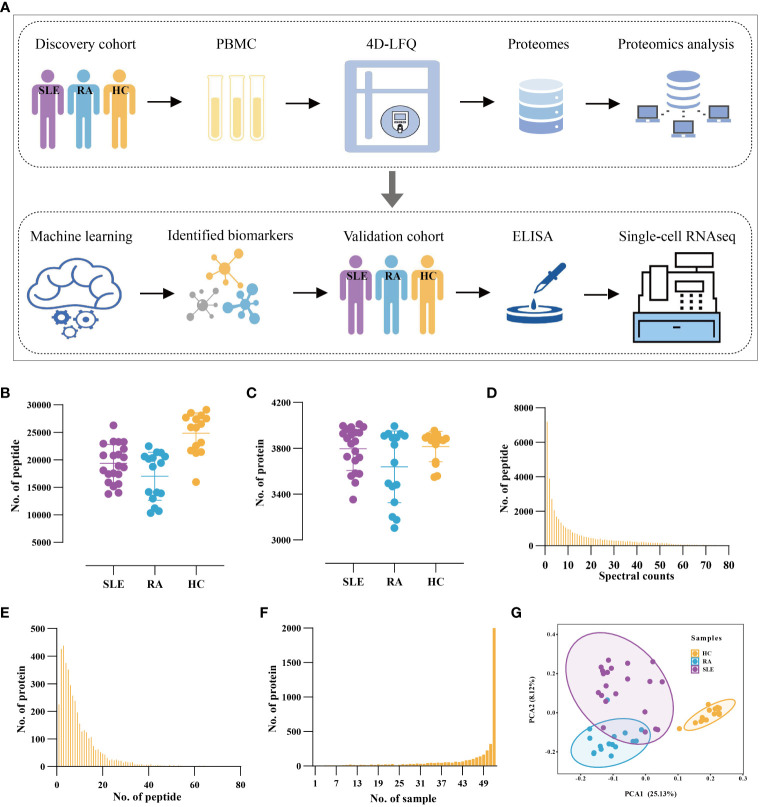
Proteomic Profiling of PBMC from SLE and RA patients and Health Volunteers. **(A)** The workflow of the study. All PBMC samples were used for 4D-LFQ proteomics analysis and ELISA analysis. **(B, C)** The distribution of numbers of quantified **(B)** peptides and **(C)** proteins in the 52 PBMC samples from three groups. Color dots represent multiple independent samples, SLE (n = 21), RA (n = 16), HC (n = 15). **(D)** The distribution of MS/MS spectral counts of quantified peptides. **(E)** The distribution of peptide numbers of quantified proteins. **(F)** The distribution of protein numbers in PBMC samples. **(G)** PCA of proteomic data in HC, SLE, and RA.

Blood samples from two cohorts of subjects were used in this study, including a discovery cohort for the 4D-LFQ screen and a validation cohort for the ELISA test. Consecutive patients diagnosed with SLE according to the 2019 EULAR/ACR classification criteria for SLE classification were recruited, regardless of disease activity. Lupus disease activity was defined according to the SLEDAI-2k score, of which active patients with SLE are SLEDAI > 4 and inactive ones are SLEDAI ≤ 4. Age-, sex-, and ethnicity-matched healthy control (HC) donors and rheumatoid arthritis (RA) patients were recruited for the study, in which HCs had no history of cancer, cardiovascular diseases, autoimmune diseases, or known infectious diseases, and RAs satisfied the 2010 EULAR/ACR classification criteria (12). The subjects were divided into four groups: active SLE (SLE_A, n=68), inactive SLE (SLE_I, n=102), HC (n=110), and RA (n=116). The workflow of the study is shown in **Figure 1A**.

### Processing blood sample

All blood samples were collected in EDTA tubes (BD Vacutainer). PBMCs were isolated *via* Ficoll gradient and stored at − 80°C prior to the protein extraction step. Total protein was extracted and digested into peptides.

### 4D-LFQ proteomics analysis

A discovery cohort was used for 4D-LFQ analysis, as described in **Tables 1** and **2**. The PBMC protein was mixed, and 52 samples were obtained (SLE_A=7; SLE_I=14; RA=16; HC=15). The peptides were dissolved in 0.1% formic acid (solvent A) and directly loaded onto a standardized bore column with C18 resin (15 cm×75 μm i.d.). In 90-min experiments, peptides were separated with a linear gradient from 6% to 24% solvent B (0.1% formic acid in 98% acetonitrile) within 70 min, followed by an increase to 35% solvent B within 14 min and further to 80% solvent B within 3 min, then holding at 80% solvent B for the last 3 min, all at a constant flow rate of 450 nL/min on a NanoElute^®^ nanoflow ultra-high-pressure liquid chromatography (UHPLC) system.

**Table 1:** Clinicopathologic characteristics of the SLE patients from discovery cohort.

Discovery cohort	SLE_A (n = 48)	SLE_I (n = 82)
Age, (mean ± SD)	37 ± 13	40 ± 12
Sex, Female (%)	40 (83.3)	70 (85.3)
Clinical Criteria			
Rash (%)	14 (29.2)	18 (22.0)
Oral ulcers (%)	3 (6.3)	0
Nonscarring alopecia (%)	3 (6.3)	2 (2.4)
Fever (%)	14 (29.2)	4 (4.9)
Serositis (%)	2 (4.2)	3 (3.7)
Synovitis involving two or more joints (%)	19 (39.6)	8 (9.8)
Renal disorder (%)	32 (66.7)	14 (17.1)
Neurologic disorder (%)	0	0
Leukopenia (< 3000/mm^3^, (%))	4 (8.3)	7 (8.5)
Thrombocytopenia (<100,000/mm^3^, (%))	6 (12.5)	2 (2.4)
LAC, (mean ± SD)	1.2 ± 0.2	1.3 ± 0.3
APTT, (sec, mean ± SD)	33.6 ± 10.4	30.9 ± 8.0
PT, (sec, mean ± SD)	12.0 ± 1.3	12.1 ± 1.7
ESR, (mm/h, mean ± SD)	40.3 ± 28.9	32.8 ± 29.1
CRP, (mg/L, mean ± SD)	9.0 ± 15.7	8.4 ± 17.8
Immunological Criteria			
Positive ANA (%)	42 (87.5)	66 (80.5)
Anti-dsDNA (%)	31 (64.6)	26 (31.7)
Anti-β2GPI, (AU/ml, mean (range))	8.5 (2.0-81.0)	11.5 (2.0-200.0)
ACL-IgG, (GPLU/ml, mean (range))	11 (1.7-120.0)	11.8 (1.0-200.0)
ACL-IgM, (MPLU/ml, mean (range))	4.6 (2.0-41.5)	2.9 (2.0-12.6)
Low complement 3 (low C3) (%)	29 (60.4)	22 (26.8)
Low complement 4 (low C4) (%)	22 (45.8)	25 (30.5)
Current drug use			
Prednison (%)	20 (41.7)	39 (47.6)
Methylprednisolone (%)	24 (50.0)	27 (32.9)
Hydroxychloroquine (%)	36 (75.0)	66 (80.5)
Ciclosporin (%)	4 (8.3)	9 (11.0)
methotrexate (%)	2 (4.2)	10 (12.2)
Mycophenolate mofetil (%)	6 (12.5)	8 (9.7)
Oral anticoagulant (%)	3 (6.3)	4 (4.9)
Aspirin (%)	9 (18.8)	9 (11.0)

All included SLE patients were detected positive ANA at least one time to satisfy 2019 EULAR/ACR SLE classification criteria. While SLE blood samples collecting, ANA of some SLE patients may turn negative. SD, standard deviation; LAC, lupus anticoagulant; APTT, activated partial thromboplastin time; PT, prothrombin time; ESR, erythrocyte sedimentation rate; CRP, C-reactive protein; ANA, anti-nuclear antibody; Anti-dsDNA, anti-double strand DNA; Anti-β2GPI, anti-β2 glycoprotein I; ACL-IgG, anticardiolipin antibody-IgG; ACL-IgM, anticardiolipin antibody-IgM; C3, complement 3; C4, complement 4.

**Table 2:** Clinicopathologic characteristics of the RA patients and HC donors from discovery cohort.

Discovery cohort		RA (n = 96)	HC (n = 90)
Age, (mean ± SD)		51 ± 15	39 ± 11
Sex, Female (%)		82 (85.4)	76 (84.4)
Disease duration years, (mean ± SD)		10.0 ± 8.7	
SJC, (mean (range))		4 (0-28)	
TJC, (mean (range))		6 (0-28)	
ESR, (mm/hour, (mean ± SD))		35.6 ± 27.2	
CRP, (mg/L, (mean ± SD))		21.2 ± 31.9	
RF positive (%)		47 (49.0)	
CCP positive (%)		48 (50.0)	
DAS28 score, (mean ± SD)		4.2 ± 1.6	
ANA positive (%)		15 (15.6)	
Low C3 (%)		3 (3.1)	
Low C4 (%)		7 (7.3)	
IGA, (g/L, (mean ± SD))		2.8 ± 1.2	
IGG, (g/L, (mean ± SD))		14.7 ± 5.1	
IGM, (g/L, (mean ± SD))		1.2 ± 0.6	
Current drug use			
Oral glucocorticoid treatment (%)	32 (33.3)		
DMARD (%)		45 (46.9)	
Oral anticoagulant (%)		2 (2.1)	
Aspirin (%)		3 (3.1)	
Biologics (%)		6 (6.3)	
Tripterygium glycosides (%)		15 (15.6)	

SD, standard deviation; SJC, swollen joint count; TJC, tender joint count; ESR, erythrocyte sedimentation rate; CRP, C-reactive protein; RF, rheumatoid factor; CCP, anti-cyclic citrullinated peptide antibody; DAS28, disease activity score (28-joint count); ANA, anti-nuclear antibody; C3, complement 3; C4, complement 4; IGA, immunoglobulin A; IGG, immunoglobulin G; IGM, immunoglobulin M; DMARD, disease modifying antirheumatic drug.

The peptides were subjected to capillary ion source ionization followed by timsTOF Pro mass spectrometry (Bruker) for analysis. The electrospray voltage applied was 1.6 kV, and the TOF was scanned for precursor and fragment ions. The MS spectra were recorded from 100 m/z to 1700 m/z, and the MS was operated in the PASEF mode. After the first stage of MS collection, a 10 times PASEF MS/MS scan was acquired for the secondary level of MS, in which the charge number of the precursor ions ranged from 0 to 5. To avoid rescans of precursor ions, the dynamic exclusion time for tandem MS scanning was set to 30 s.

### 4D-LFQ proteomics data analysis

The resulting MS/MS data were processed using MaxQuant search engine (v.1.6.6.0). Tandem mass spectra were searched against the human UniProt database (Homo_sapiens_9606_SP_20191115, 20380 entries), and a reversed sequence library was employed to control the false discovery rate (FDR) at less than 1% for peptide spectrum matches and protein group identifications. The missing cleavages allowed up to two, and the required minimum peptide sequence length was seven amino acids. Carbamidomethylation of Cys residues was regarded as a fixed modification, and acetylation of protein N termini and oxidation of Met residues as variable modifications. The mass tolerance for precursor ions was set as 20 ppm in the first and main searches, and the mass tolerance for fragment ions was set as 0.02 Da.

### Proteomic data normalization and imputation

For each sample of the PBMC proteomics data, the intensity of a protein in one sample was normalized against the average intensity of the protein in all samples to obtain the relative protein intensity, which was used for further analysis. To ensure data quality and maximize the use of proteomic data, proteins quantified in < 60% of the samples were discarded. To impute missing values of the remaining proteins, we used the R package knnImputation function.

### Heatmap analysis

A visual analysis module in TBtools (13) was used to execute heatmap analysis for PBMC proteomics data from SLE, RA, and HC. Heatmap analysis was also performed for proteins identified in profile 10 using TBtools.

### GSVA and GSEA analysis

All the gene sets were downloaded from the MSigDB database. Gene Set Variation Analysis (GSVA) was utilized to analyze the Kyoto Encyclopedia of Genes and Genomes (KEGG) pathways of differentially expressed proteins (DEPs) using the R package GSVA and GSVAdata. The selection criteria for significantly enriched KEGG pathways were set at *P* < 0.05. The selection criteria for the differentially activated KEGG pathways were based on |t | > 2. GSEA was used to predict the differentially enriched KEGG pathways between SLE and HC or RA using the R package GSEABase. |Normalized enrichment score (NES)|> 1, *P* < 0.05, and FDR < 0.25 were set as the cutoffs.

### Short time-series expression minor analysis

To identify the molecular signatures associated with SLE disease exacerbation, short time-series expression minor (STEM) analysis was used to cluster protein expression profiles from HC donors, SLE_I patients, and SLE_A patients (14). The expression data were normalized and the STEM clustering method was utilized. The minimum absolute expression change was set at 0.5 for molecule filtering, and the maximum correlation between any two model profiles was set at 0.9. The profiles with *P* < 0.05 based on the number of clustered genes were considered as significantly enriched clusters.

### Functional enrichment analysis for profile 10 by Metascape

Metascape pathway enrichment analysis (https://metascape.org) (15) was used for the analysis of PBMC proteins in profile 10, and the relevant parameters were as follows: minimum overlap, 3; *P* value cutoff, 0.01.

### POC-SLE for SLE diagnosis and disease exacerbation assessment

To identify biomarkers for disease diagnosis and assess disease exacerbation in SLE, we constructed a classification model named the Prioritization of Optimal biomarker Combinations for SLE (POC-SLE). First, we used the R package Random Forest to execute a random forest analysis (RFA) with 1000 bootstrap sampling iterations. We used the GINI index to identify the top 100 ranked DEPs as the first candidate biomarker selection set (CBSS). Second, we used the R package OPLS to perform Orthogonal Projections to Latent Structures-Discriminant Analysis (OPLS-DA). We used variable importance for the projection (VIP) to evaluate DEPs with VIP > 1 as the second CBSS. Finally, we took the intersection of the two CBSSs for biomarker determination and established an ROC curve to evaluate the diagnostic and prediction performance for biomarkers when used alone and in combination.

### ELISA validation

PBMCs from 80 subjects in the cross-sectional cohort were included, comprising 20 HC donors, 20 RA patients, 20 inactive SLE patients (SLEDAI ≤ 4), and 20 patients with active SLE (SLEDAI > 4), as described below (**Tables 3** and **4**). Importantly, all groups had comparable age and sex. PBMC proteins were extracted from all samples. Following manufacturer protocols, the protein biomarkers were validated using ELISA assays (name, Manufacturer, catalog number), including Anti-TOMM40, Laibio, JL13785; Anti-STAT2, Laibio, JL15296; Anti-OAS3, Laibio, JL13727; Anti-STATl, Laibio, JL15295; Anti-MXl, Laibio, JL13729; Anti-lFIT3, Laibio, JL13731; Anti-SMClA, KALANG, KL-12419H; Anti-PHACTR2, Laibio, JL13736; Anti-GOT2, Laibio, JL13749; Anti-SELL, Laibio, JL13761; Anti-CMC4, Abebio, AE32512HU; Anti-MAP2Kl, Laibio, JL13790; Anti-CMPK2, Laibio, JL13775; Anti-ECPAS, FineTest, EH15161; Anti-DTX3L, KALANG, KL-8064H; Anti-MZBl, FineTest, EH10389; Anti-SRAl, KALANG, KL-12585H. In brief, an optimal dilution of PBMC proteins was added to a microplate precoated with capture antibody, incubated, washed, followed by the addition of capture antibody, horseradish peroxidase, and substrate. The absolute levels of each PBMC protein were determined using standard curves run on each ELISA plate and normalized for analysis.

**Table 3:** Clinicopathologic characteristics of the SLE patients from validation cohort.

Validation cohort	SLE_A (n = 20)	SLE_I (n = 20)
Age, (mean ± SD)	33 ± 10	33 ± 11
Sex, Female (%)	19 (95)	19 (95)
Clinical Criteria		
Rash (%)	3 (15)	2 (20)
Oral ulcers (%)	0	0
Nonscarring alopecia (%)	0	1 (5)
Fever (%)	4 (20)	3 (15)
Serositis (%)	2 (10)	1 (5)
Renal disorder (%)	12 (60)	1 (5)
Neurologic disorder (%)	0	0
Leukopenia (< 3000/mm^3^, (%))	6 (30)	1 (5)
Thrombocytopenia (<100,000/mm^3^, (%))	4 (20)	0
LAC, (mean ± SD)	1.3 ± 0.5	1.1 ± 0.1
APTT, (sec, mean ± SD)	34.5 ± 10.4	31.5 ± 3.7
PT, (sec, mean ± SD)	11.7 ± 1.1	12.0 ± 1.7
ESR, (mm/h, mean ± SD)	39.2 ± 32.7	12.6 ± 9.3
CRP, (mg/L, mean ± SD)	8.3 ± 9.3	5.4 ± 10.4
Immunological Criteria		
Positive ANA (%)	20 (100)	15 (75)
Anti-dsDNA (%)	13 (65)	6 (30)
Anti-β2GPI, (AU/ml, mean (range))	6.6 (1.6-46.8)	5.7 (1.6-23.3)
ACL-IgG, (GPLU/ml, mean (range))	6.2 (1.4-32.2)	5.1 (1.0-18.6)
ACL-IgM, (MPLU/ml, mean (range))	4.0 (1.5-22.5)	3.0 (2-7.4)
Low C3 (%)	19 (95)	8 (40)
Low C4 (%)	17 (85)	8 (40)
Current drug use		
Prednison (%)	9 (45)	7 (35)
Methylprednisolone (%)	10 (50)	11 (55)
Hydroxychloroquine (%)	16 (80)	19 (95)
Ciclosporin (%)	4 (20)	1 (5)
Methotrexate (%)	0	3 (15)
Mycophenolate mofetil (%)	5 (25)	3 (15)
Oral anticoagulant (%)	0	0
Aspirin (%)	5 (25)	1 (5)

All included SLE patients were detected positive ANA at least one time to satisfy 2019 EULAR/ACR SLE classification criteria. While SLE blood samples collecting, ANA of some SLE patients may turn negative. SD, standard deviation; LAC, lupus anticoagulant; APTT, activated partial thromboplastin time; PT, prothrombin time; ESR, erythrocyte sedimentation rate; CRP, C-reactive protein; ANA, anti-nuclear antibody; Anti-dsDNA, anti-double strand DNA; Anti-β2GPI, anti-β2 glycoprotein I; ACL-IgG, anticardiolipin antibody-IgG; ACL-IgM, anticardiolipin antibody-IgM; C3, complement 3; C4, complement 4.

**Table 4:** Clinicopathologic characteristics of the RA patients and HC donors from validation cohort.

Discovery cohort	RA (n=20)	HC (n=20)
Age, (mean ± SD)	37 ± 9	33 ± 9
Sex, Female (%)	19 (95)	19 (95)
Disease duration years, (mean ± SD)	6.1 (0.25-20)	
SJC, (mean (range))	4 (0-20)	
TJC, (mean (range))	5 (0-20)	
ESR, (mm/hour, (mean ± SD))	32.8 ± 27.0	
CRP, (mg/L, (mean ± SD))	11.0 ± 15.9	
RF positive (%)	11 (55)	
CCP positive (%)	12 (60)	
DAS28 score, (mean ± SD)	3.9 ± 1.4	
ANA positive (%)	6 (30)	
Low C3 (%)	4 (20)	
Low C4 (%)	6 (30)	
IGA, (g/L, (mean ± SD))	2.5 ± 1.0	
IGG, (g/L, (mean ± SD))	16.3 ± 8.2	
IGM, (g/L, (mean ± SD))	1.4 ± 0.6	
Current drug use		
Oral glucocorticoid treatment (%)	9 (45)	
DMARD (%)	19 (95)	
Oral anticoagulant (%)	0	
Aspirin (%)	0	
Biologics (%)	0	
Tripterygium glycosides (%)	1 (5)	

SD, standard deviation; SJC, swollen joint count; TJC, tender joint count; ESR, erythrocyte sedimentation rate; CRP, C-reactive protein; RF, rheumatoid factor; CCP, anti-cyclic citrullinated peptide antibody; DAS28, disease activity score (28-joint count); ANA, anti-nuclear antibody; C3, complement 3; C4, complement 4; IGA, immunoglobulin A; IGG, immunoglobulin G; IGM, immunoglobulin M; DMARD, disease modifying antirheumatic drug.

### PBMC single-cell RNA sequencing for SLE

We applied single-cell RNA sequencing (scRNA-seq) to PBMCs from patients with SLE and HC donors (16). In addition, publicly available scRNA-seq data from patients with active SLE, inactive SLE, and healthy controls were obtained from the Gene Expression Omnibus (GEO) database under accession numbers GSE135779 (17), GSE142016 (18), GSE142637 (19), and GSE162577 (20). For all datasets, post-quality control expression datasets contained PBMC, yielding a total of 400,510 cells from 21 healthy controls and 46 SLE patient biopsies.

All datasets were integrated and normalized expression values were obtained using the FindIntegrationAnchors and IntegrateData functions, and then the whole expression data and variable genes were scaled and identified using the ScaleData and FindVariableFeatures functions, respectively. rincipal component analysis (PCA) was applied to genes from the selected cells. The first 25 PCs were used for uniform manifold approximation and projection (UMAP) analysis. Then, we used the FindClusters function that implements shared nearest neighbor (SNN) modularity optimization based clustering algorithm on 25 PCA components with resolution 0.1 - 1.0 leading to 10-24 clusters, and a resolution of 0.4 was chosen for further analysis; to identify marker genes, the FindAllMarkers function was used with likelihood-ratio test for single cell gene expression (21). We performed differentially expressed gene (DEG) analysis by comparing each cluster between SLE and HC using the Wilcoxon rank sum test, and genes with *P* < 0.05 were designated as a significant signature. We also performed differentially expressed gene (DEG) analysis by comparing each cluster between inactive SLE (SLEDAI ≤ 4) and active SLE (SLEDAI > 4) using the Wilcoxon rank sum test, and genes with *P* < 0.05 were designated as significant signatures.

### Statistics analyses

All statistical analyses were performed using Prism (GraphPad, v.8.2.1) and R software (v.4.1.0). Altered proteins with *P* < 0.05, FC > 1.5 or < 0.67 were considered differentially expressed proteins. Spearman’s correlation was used to describe the relationships between the clinical parameters and proteins. Statistical significance was assessed using unpaired two-tailed Student’s t-test, Mann–Whitney U test, moderated t-test, permutation test, likelihood-ratio test, or Wilcoxon rank-sum test, where appropriate.

## Results

### Proteomics profiling of PBMCs from SLE patients

From the discovery cohort based on 4D-LFQ technology, we obtained a total of 41,263 peptides from 21 SLE, 16 RA, and 15 HC samples (**Figure 1B**). The peptides were mapped to corresponding protein sequences and 4247 proteins were identified in 52 samples, with average number of proteins ranging from 3,638 to 3,815 in the three groups (**Figure 1C**). To assess the reliability of proteomic profiling, we found that 33,422 peptides (80.9%) were matched by ≥ 2 spectral counts (**Figure 1D**), with an average spectral count of 17 for all peptides, indicating the reliability of proteomic data at the peptide level. We discovered that 4,043 proteins (95.2%) could be hunted by ≥2 peptides, and the average peptides were calculated to be 10 for all proteins (**Figure 1E**), implying high reliability at the protein level. We also analyzed the distribution of proteins in different samples and found that up to 1999 proteins (47.1%) were concurrently quantified in all 52 samples (**Figure 1F**), indicating high repeatability of the proteomic data for the discovery cohort.

**Figure 2:**
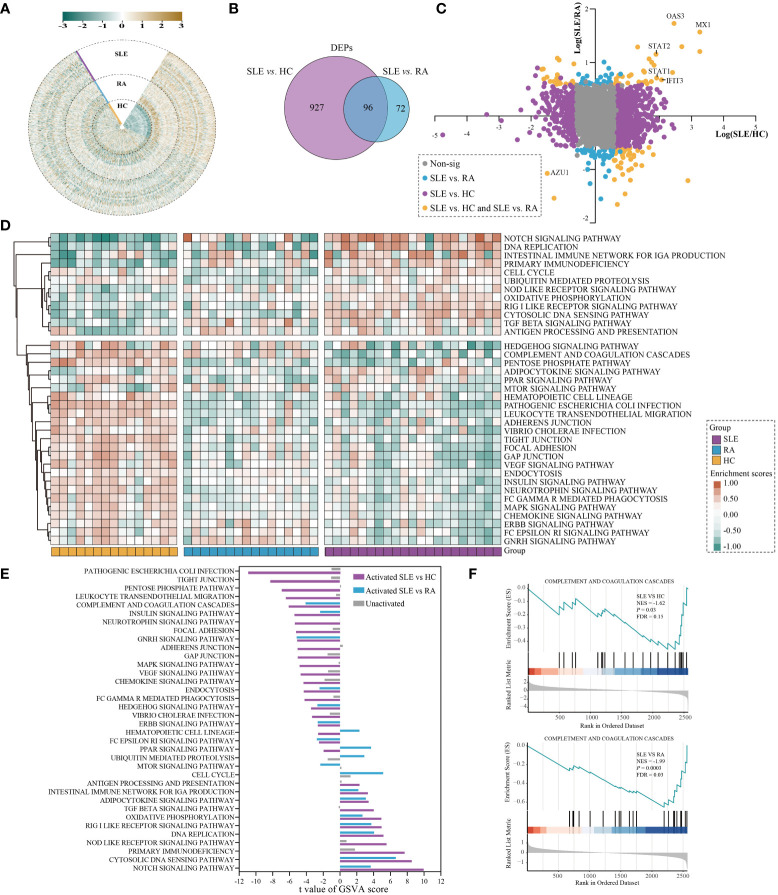
Differential analysis in protein expression levels between SLE and HC or RA. **(A)** The heatmap for the expression of PBMC proteins in SLE, RA, and HC. The expression of each proteins was normalized by Z score normalization. **(B)** Venn diagram summarising the differential and overlapping proteins between SLE and HC or RA (fold change(FC) > 1.5 or < 0.67, unpaired two-sided Student’s t-tests, *P* < 0.05). **(C)** Plots of fold changes of differentially expressed proteins in SLE *vs.* RA only, SLE *vs*. HC only, and SLE *vs*. both. **(D)** The enrichment score for 36 KEGG pathways by GSVA in SLE, RA, and HC. The brown and green nodes represent upregulation and downregualtion state of pathway, respectively (moderated t-test, *P* < 0.05). **(E)** The differentially activated KEGG pathways between SLE *vs.* RA and SLE *vs.* HC. The blue and purple bands represent the activated pathways of SLE *vs.* RA and SLE *vs.* HC, respectively; and the gray bands represent the unactivated pathway. **(F)** GSEA of complement and coagulation cascades gene sets were significantly differentially enriched between SLE and HC or RA (permutation test, *P* < 0.05).

After the database search, the LFQ intensity was normalized to obtain the relative quantification value of each protein. According to previous studies (22), it is credible to include proteins having less than 50% missing data to ensure that each sample had enough data for imputation. Furthermore, to discover the efficiency biomarkers as far as possible under the condition of reliable data, we retained the proteins that were expressed in 60% to 100% samples of the large discovery cohort. To ensure high data quality, only 2602 proteins mutually quantified in > 60% of the samples (≥ 32) were reserved for the discovery cohort. For each protein, K-Nearest Neighbor (KNN) was applied to impute the missing values. The PCA of the 52 samples was performed using 2602 proteins with normalized expression values (**Figure 1G**). The SLE and RA samples were not completely separated; consistent with this observation, SLE and RA patients presented certain overlapping manifestations (23). The normalized expression of 2602 proteins in the SLE, RA, and HC groups was visualized in the heatmap (**Figure 2A**). A substantial number of proteins were differentially expressed in different PBMC samples from the three groups.

### PBMC proteomics alternations of SLE patients

Using the PBMC proteomic data, we identified signatures of SLE which underwent significant differential expression in SLE samples compared to RA and HC subjects. In total, 1023 and 168 differentially expressed proteins (DEPs) were found between SLE *vs.* HC and SLE *vs.* RA in PBMC samples, respectively (**Figure 2B** and **Table S1**, fold change (FC) > 1.5 or < 0.67, unpaired two-sided Student’s t-tests, *P* < 0.05). This indicated that the alterations of PBMC in SLE became less extensive in different autoimmune rheumatic disease conditions compared with healthy donors. The fold-changes of proteins in SLE *vs*. HC, SLE *vs*. RA, and SLE *vs*. both are highlighted in **Figure 2C**. The interferon-induced GTP-binding protein Mx1 (MX1), 2’-5’-oligoadenylate synthase 3 (OAS3), and interferon-induced protein with tetratricopeptide repeats 3 (IFIT3), which are important in the type I interferon signaling pathway (24–26), were significantly upregulated in SLE *vs*. both, with MX1 being the most upregulated protein. Signal transducer and activator of transcription 1 (STAT1) and STAT2, which participate in JAK/STAT signaling in SLE (27), were also notably upregulated in SLE *vs.* both. Azurocidin (AZU1), which plays a role in inflammatory and cytokine stimulus responses (28), is prominently downregulated in SLE.

The DEPs were then subjected to differentially enriched pathway analysis between SLE and HC or RA by Gene Set Variation Analysis (GSVA) (29). In total, 181 KEGG pathways were annotated in the three groups; 107 and 59 KEGG pathways were significantly differentially enriched in SLE *vs.* HC and SLE *vs.* RA, respectively (**Table S2**, moderated t-test, *P* < 0.05). Classification of these KEGG pathways revealed that cell processes (32.0%), metabolic processes (22.7%), disease-related (16.6%), and signaling (14.4%) accounted for the highest proportion (**Figure S1** and **Table S2**). The heatmap shows the enrichment scores of 36 KEGG pathways in each sample of the three different groups after processing by GSVA, including signaling, infection process, and cell process (**Figure 2D**, moderated t-test, *P* < 0.05). KEGG pathways with |t value| > 2 further showed the differentially activated pathways in SLE *vs.* HC and SLE *vs.* RA (**Figure 2E**). Compared with RA and HC, PBMC in SLE were mainly activated in the NOTCH signaling pathway, cytosolic DNA sensing pathway, DNA replication, RIG I-like receptor signaling pathway, oxidative phosphorylation, adipocytokine signaling pathway, and the intestinal immune network for IgA production. In contrast, compared with RA and HC, PBMC in SLE were mainly inhibited in the complement and coagulation cascades, insulin signaling pathway, GNRH signaling pathway, endocytosis, HEDGEHOG signaling pathway, ERBB signaling pathway, and Fc-epsilon RI signaling pathway. These results are consistent with previous reports showing that inflammation and immunity are associated with SLE (30, 31). Gene Set Enrichment Analysis (GSEA) (32) was used to assess differentially enriched pathways between patients with SLE and controls, as shown in **Table S3**. We also found that complement and coagulation cascades from GSEA were suppressed in SLE (normalized enrichment score, NES, SLE *vs.* HC_NES = -1.62; SLE *vs.* RA_NES = -1.99; **Figure 2F**, permutation test, *P* < 0.05).

### Proteomics alterations associated with disease exacerbation of SLE patients

**Figure 3:**
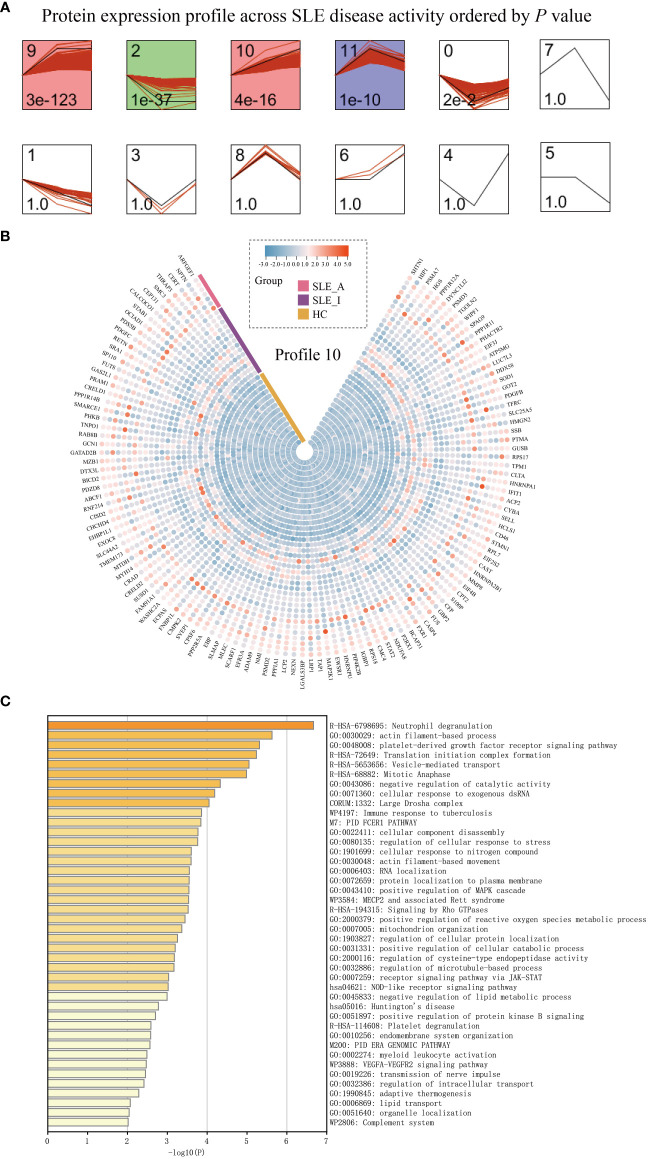
PBMC proteomic data for SLE disease exacerbation. **(A)** PBMC proteomics profiles of STEM analysis. STEM analysis was applied to obtain the protein expression profiles across HC, SLE_I, and SLE_A. Profile ID was shown at the top left corner of the profile, and significance (*P* value) was shown at the bottom left corner of the profile. Red lines in each profile represent the expression pattern of proteins across HC, SLE_I, and SLE_A (permutation test, *P* < 0.05). **(B)** Heatmap for the expression of proteins in profile 10 along with disease exacerbation. **(C)** The function analysis of profile 10 in Metascape platform (*P* < 0.01).

To understand how PBMC protein expression changes with SLE disease exacerbation, we applied a Short Time-series Expression Miner (STEM) analysis (14) for active SLE (SLE_A), inactive SLE (SLE_I), and HC to obtain different profiles of protein expression behavior. We identified five significant protein profiles with different expression behaviors across the HC, SLE_I, and SLE_A groups, including profiles 0, 2, 9, 10, and 11 (**Figure 3A**, permutation test, *P* < 0.05). The expression of PBMC proteins in profile 10 increased with an increase in SLEDAI score, suggesting a positive correlation (**Figure 3B**). The PBMC proteins in profile 10 were significantly enriched in neutrophil degranulation, the JAK-STAT signaling pathway, and the complement system on the Metascape platform (**Figure 3C**, *P* < 0.01).

### Machine learning based selection of biomarker combinations for SLE disease diagnosis and disease exacerbation assessment

**Figure 4:**
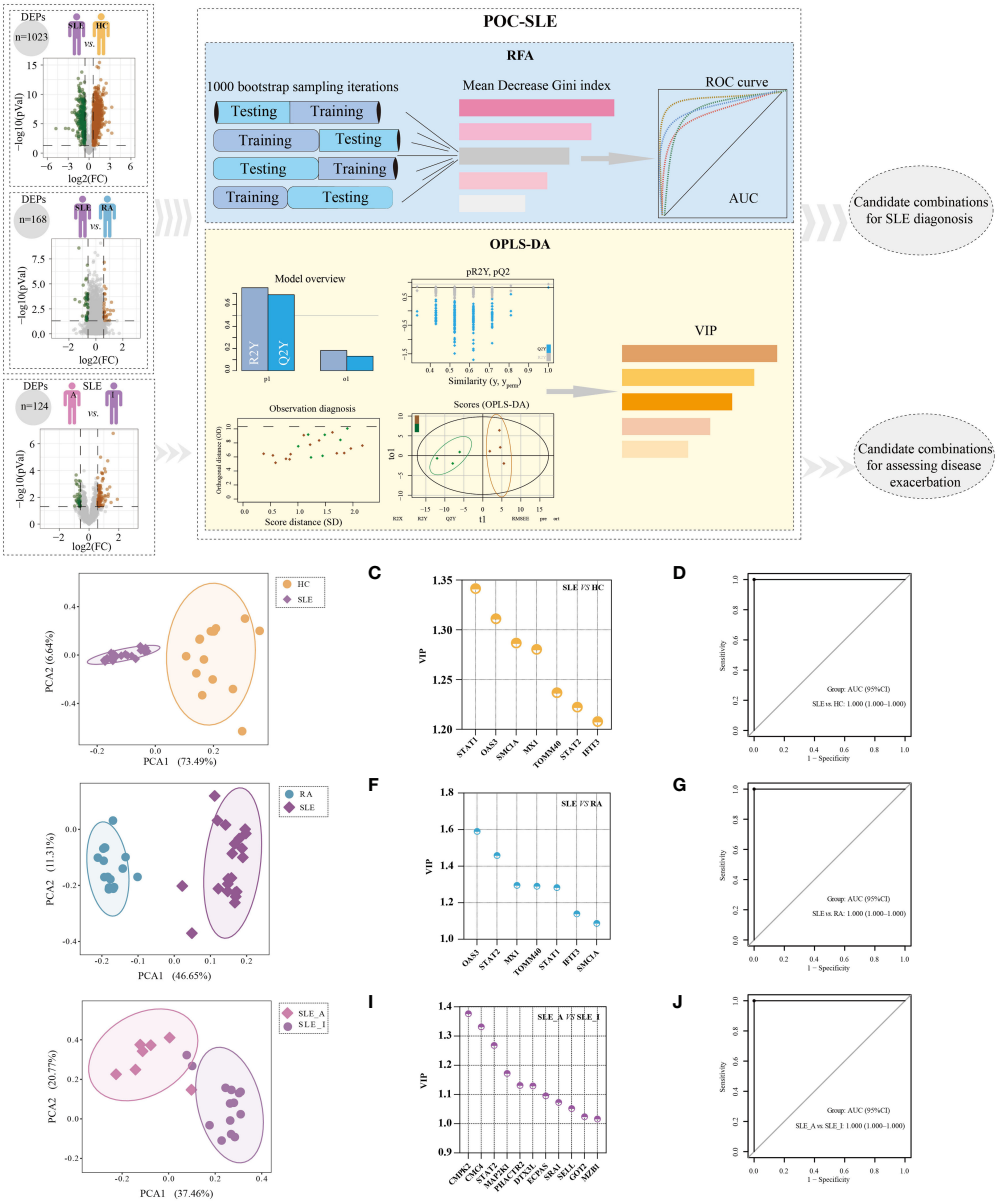
Identification of potential biomarker combinations for the disease diagnosis and assessing disease exacerbation of SLE patients. **(A)** The workflow of POC-SLE, including 1000 bootstrap sampling iterations RFA and OPLS-DA. The PCA plot for distinguishing SLE and HC **(B)**, SLE and RA **(E)**, and SLE_A and SLE_I **(H)**. The VIP value of potential biomarker combinations for discriminating SLE and HC **(C)**, SLE and RA **(F)**, SLE_A and SLE_I **(I)**. ROC curve of the biomarker combination for disease diagnosis to differentiate SLE and HC **(D)**, SLE and RA **(G)**; ROC curve of the biomarker combination for assessing disease exacerbation to distinguish SLE_A and SLE_I **(J)**.

In light of the PBMC proteomics data, we applied a new machine-learning pipeline named Prioritization of Optimal biomarker Combinations for SLE (POC-SLE) to identify potential biomarker combinations for SLE diagnosis to discriminate SLE patients from HC donors and RA cases. The POC-SLE consists of three steps, including 1000 bootstrap sampling iterations Random Forest Analysis (RFA) to select the top 100 ranked DEPs as the first candidate biomarker selection set (CBSS). Then, Orthogonal Projections to Latent Structures-Discriminant Analysis (OPLS-DA) was used to obtain variable importance for the projection (VIP) > 1 DEPs as the second CBSS. After that, the intersection of two sets were taken for biomarker determination (**Figure 4A**). The identified CBSSs are shown in **Table S4**, including SLE *vs.* HC and SLE *vs.* RA. PCA was performed to evaluate the reliability of the POC-SLE pipeline; it showed that the SLE and HC samples were clearly classified into different groups by biomarker determination (**Figure 4B**), indicating the reliability of the machine-learning strategy for distinguishing patients with SLE and HC. The clustering results for SLE and RA are shown in **Figure 4E**. We then used the intersection of biomarker determination from SLE *vs*. HC and SLE *vs*. RA and obtained seven proteins that could be used as a final biomarker combination for SLE diagnosis, including IFIT3, MX1, OAS3, STAT1, STAT2, mitochondrial import receptor subunit TOM40 homolog (TOMM40), and structural maintenance of chromosome protein 1A (SMC1A). The VIP values of the seven proteins in SLE *vs*. HC and SLE *vs*. RA are shown in **Figures 4C, F**, respectively. The RFA AUC value of this seven-protein combination to distinguish the SLE and control groups was calculated as 1 (SLE *vs*. HC, 95% confidence interval [CI] = 1–1) and 1 (SLE *vs*. RA, 95% CI = 1–1) (**Figures 4D, G**, respectively). Moreover, the AUC values of each of the seven proteins ranged from 0.827 to 1, differentiating SLE from HC and RA (**Figures S2A, B**), indicating that even when used alone, these proteins could be used to distinguish between different groups under most conditions.

To identify the potential biomarker combination for assessing SLE disease exacerbation, we also constructed POC-SLE to discriminate SLE_A subjects from SLE_I cases. We identified two CBSSs shown in **Table S4**. PCA also showed that the cluster variations between the SLE_A and SLE_I samples were clear (**Figure 4H**). We identified 53 proteins as biomarkers for distinguishing SLE_A from SLE_I by POC-SLE, then took the intersection between biomarker determination and profile 10 subset (**Table S5**), and obtained 11 continuously increased expression final biomarker combinations for assessing SLE disease exacerbation, including phosphatase and actin regulator 2 (PHACTR2), glutamate oxaloacetate transaminase 2 (GOT2), L-selectin (SELL), Cx9C motif-containing protein 4 (CMC4), dual specificity mitogen-activated protein kinase kinase 1 (MAP2K1), cytidine/uridine monophosphate kinase 2 (CMPK2), Ecm29 proteasome adaptor and scaffold (ECPAS), Deltex E3 ubiquitin ligase 3 L (DTX3L), marginal zone B and B1 cell specific protein (MZB1), steroid receptor RNA activator 1 (SRA1), and STAT2. The VIP values of the 11 proteins in SLE_A *vs*. SLE_I are shown in **Figure 4I**. The RFA AUC value of the 11-protein combination to distinguish SLE_A from SLE_I was calculated as 1 (95% CI = 1–1) (**Figure 4J**). Moreover, the AUC values for each of the 11 proteins ranged from 0.786 to 0.970 (**Figure S2C**), indicating that even when used alone, these proteins could discriminate between SLE_A and SLE_I.

### Validation of biomarker combinations for SLE disease diagnosis and assessing SLE disease exacerbation

To validate the veracity of the machine learning-based disease diagnosis and assessment of disease exacerbation in SLE patients, we collected 80 PBMC samples as a new cohort (validation cohort) for ELISA validation, comprising of 20 SLE_A, 20 SLE_I, and 20 RA patients, together with 20 HC volunteers. The demographic characteristics, clinical features, and pharmacotherapy history of these patients are presented in **Tables 3** and **4**.

First, we succeeded in detecting 14 proteins, but three proteins, SMC1A, DTX3L, and MZB1, were not detected by ELISA. We found that the ELISA results of six proteins for SLE disease diagnosis confirmed the proteomics data obtained in our study. ELISA results showed that PBMC IFIT3 (**Figure S3A**; mean SLE 57ng/mL, mean RA 42ng/mL, *P* = 0.002), MX1 (**Figure S3B**; mean SLE 117ng/mL, mean RA 83ng/mL, *P =* 0.003), TOMM40 (**Figure S3C**; mean SLE 56ng/mL, mean RA 35ng/mL, *P* < 0.0001), STAT1 (**Figure S3D**, mean SLE 5,412pg/mL, mean RA 3,634pg/mL, *P* = 0.0004), and STAT2 (**Figure S3E**; mean SLE 2.4ng/mL, mean RA 1.2ng/mL, *P* = 0.002), and OAS3 (**Figure S3F**, mean SLE 1,214 nmol/L, mean RA 847 nnmol/L, *P* = 0.006) levels were significantly upregulated in SLE patients compared to those in RA patients. Furthermore, PBMC IFIT3 (**Figure S3A**; mean HC 47ng/mL, *P* = 0.04), MX1 (**Figure S3B**, mean HC 90ng/mL, *P* = 0.02), TOMM40 (**Figure S3C**; mean SLE 56ng/mL, mean RA 35ng/mL, *P* < 0.0001), and STAT1 (**Figure S3D**, mean HC 4,214pg/mL, *P* = 0.02) levels were significantly elevated in SLE patients compared to those in HC individuals. And STAT2 (**Figure S3E**; mean HC 1.8ng/mL, *P* > 0.05) and OAS3 (**Figure S3F**, mean HC 999 nmol/L, *P* > 0.05) levels were both slightly higher in the SLE group than in the HC group. Moreover, we found that the ELISA results of nine proteins for assessing SLE disease exacerbation confirmed the proteomic data. ELISA results showed that PBMC PHACTR2 (**Figure S4A**; mean SLE_A 458ng/mL, mean SLE_I 452ng/mL), GOT2 (**Figure S4B**; mean SLE_A 49ng/mL, mean SLE_I 47ng/mL), L-selectin (**Figure S4C**; mean SLE_A 17ng/mL, mean SLE_I 16ng/mL), CMC4 (**Figure S4D**, the mean SLE_A 0.24ng/mL, mean SLE_I 0.21ng/mL), MAP2K1 (**Figure S4E**, mean SLE_A 18ng/mL, mean SLE_I 16ng/mL), CMPK2 (**Figure S4F**; mean SLE_A 14ng/mL, mean SLE_I 13ng/mL), ECPAS (**Figure S4G**; mean SLE_A, 3,516pg/mL; mean SLE_I, 3,382pg/mL), SRA1 (**Figure S4H**; mean SLE_A 4.7ng/mL, mean SLE_I 2.8ng/mL), and STAT2 (**Figure S4I**, mean of SLE_A 1.8ng/mL, mean of SLE_I 1.7ng/mL) were all slightly upregulated in active SLE (SLE_A) patients compared to the inactive SLE (SLE_I) patients.

**Figure 5:**
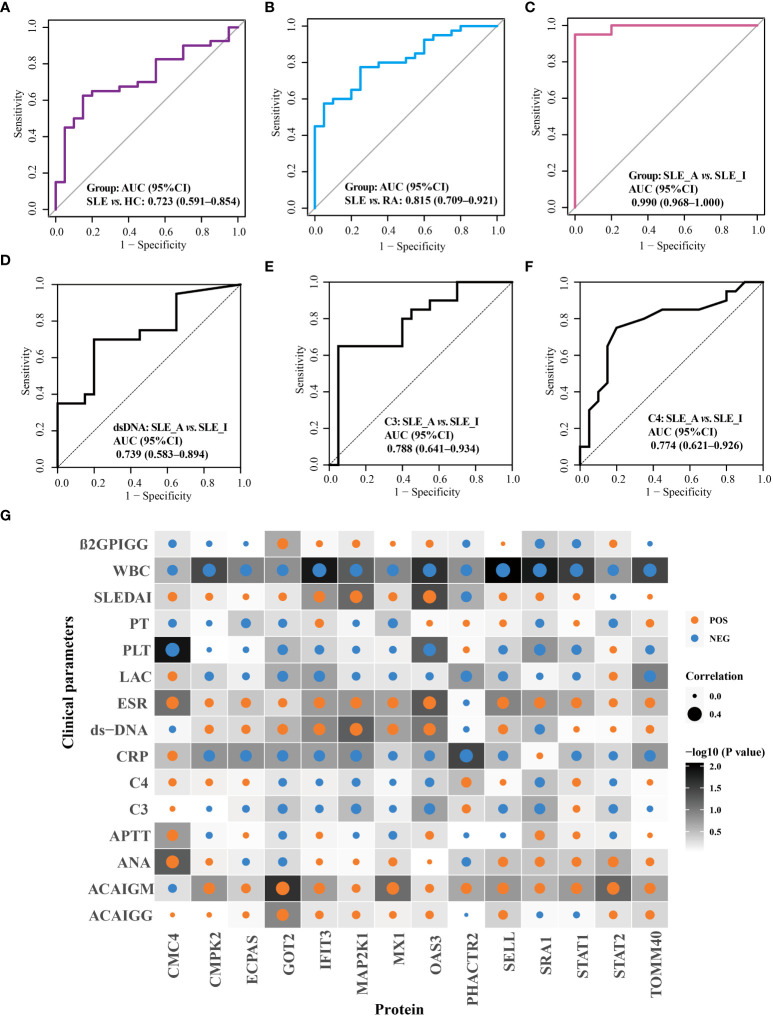
Validation of disease diagnosis and assessing disease exacerbation biomarker combinations of SLE patients. ROC curves for the disease diagnosis of distinguishing SLE from HC **(A)** and SLE from RA **(B)**. ROC curve for the assessing disease exacerbation of distinguishing SLE_A from SLE_I **(C)**. ROC curves for distinguishing SLE_A from SLE_I using anti-DNA **(D)**, C3 **(E)**, and C4 **(F)**. **(G)** Correlation plot of clinical parameters with biomarkers. Each square represents a correlation. A darker background indicates a lower *P* value, as determined by Spearman correlation. The size of the dot in each square represents the magnitude of the correlation, with a bigger dot representing higher correlation. Blue and orange dots indicate negative correlation and positive correlation, respectively.

The performance of these proteins in disease diagnosis and assessment of disease exacerbation was further highlighted using receiver operating characteristic (ROC) curves. As displayed in **Figure 5**, the combination of OAS3, IFIT3, MX1, STAT1, STAT2, and TOMM40 exhibited the disease diagnostic potential, with AUC of 0.723 (95% CI = 0.591–0.854) and 0.815 (95% CI = 0.709–0.921), in distinguishing SLE from HC and RA, respectively (**Figures 5A, B**). Next, as shown in **Figure 5C**, the combination of PHACTR2, GOT2, L-selectin, CMC4, MAP2K1, CMPK2, ECPAS, SRA1, and STAT2 exhibited potential to assess disease exacerbation, with an AUC of 0.990 (95% CI = 0.968–1), in distinguishing SLE_A from SLE_I. The nine proteins combined into a panel outperformed traditional clinical parameters, such as anti-dsDNA (AUC = 0.739), C3 (AUC = 0.788), and C4 (AUC = 0.774), in distinguishing SLE_A from SLE_I, as shown in **Figures 5D–F**. Next, the correlation between the clinical features and biomarkers was analyzed using a correlation plot, as shown in **Figure 5G**. We found that the expression levels of IFIT3, MAP2K1, and OAS3 were positively correlated with SLEDAI, indicating that these are biomarkers related to disease exacerbation. Furthermore, GOT2, IFIT3, MAP2K1, MX1, and OAS3 were positively correlated with dsDNA levels. Conversely, GOT2, IFIT3, MAP2K1, MX1, OAS3, SRA1, and STAT2 were negatively correlated with C3 and C4 levels. Thus, the identified PBMC biomarkers are strong indicators for assessing disease exacerbation in SLE patients.

### Differential expression of PBMC biomarkers in different immune cells for SLE

**Figure 6:**
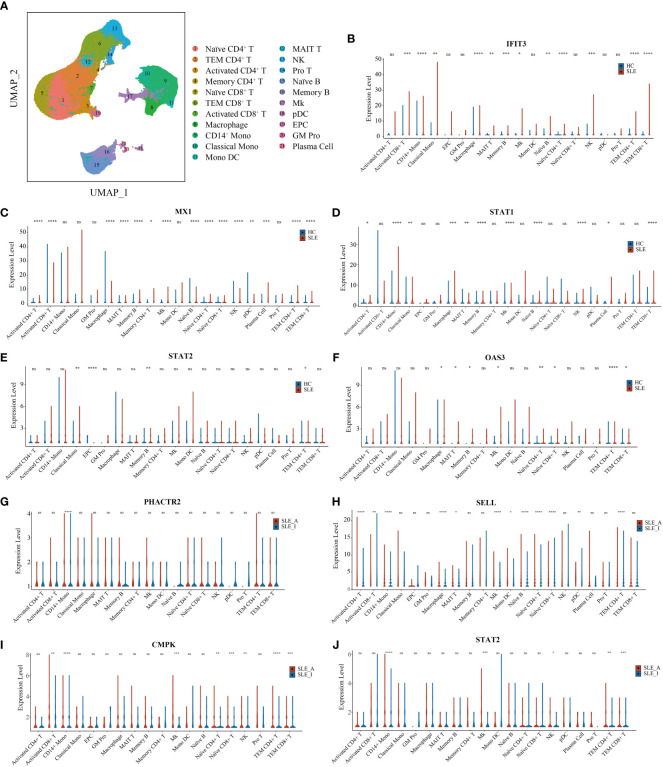
UMAP visualization for PBMC scRNAseq and Violin plots of scRNAseq data for SLE *vs*. HC and active SLE *vs*. inactive SLE. **(A)** Two-dimensional integrated UMAP visualization of PBMC cells combined from 46 SLE patient and 21 HC donors. PBMC were divided into clusters based on the expression of canonical genes. **(B-F)** Violin plots showing the differented expression profile of five SLE disease diagnosis related genes identified between SLE and HC; **(G–J)** Violin plots showing the differented expression profile of four assessing disease exacerbation related genes identified between active SLE and inactive SLE (Wilcoxon rank sum test, *P* < 0.05). Red dot presents for HC or inactive SLE, and green dot presents for SLE or active SLE. *P < 0.05, **P < 0.01, ***P < 0.001, ****P < 0.0001, ns, not significant.

To further explore the immune cell origins of PBMC biomarkers in lupus, PBMC scRNA-seq database from 46 SLE and 21 HC peripheral blood samples were analyzed. After quality control, we clustered 21 cell types, including T cells, B cells, natural killer (NK) cells, monocytes, dendritic cells (DC), megakaryocytes (Mks), granulocyte-monocyte progenitors (GM Pro), and erythroid-like and erythroid precursor cells (EPC) (**Figure 6A**). To explore the transcript expression levels of biomarkers in SLE patients, we first compared the total PBMC transcript expression of each biomarker for disease diagnosis (*IFIT3*, *MX1*, *TOMM40*, *STAT1*, *STAT2*, and *OAS3*) between the SLE and HC groups. We found that the scRNA-seq results of *IFIT3*, *MX1*, *STAT1*, *STAT2*, and *OAS3* for SLE disease diagnosis showed the same changes as the proteomics data between the SLE and HC groups in our study (**Figure S5A**, all *P* < 0.0001). We then compared the differential expression of these five genes between SLE and HC samples, as shown in violin plots (**Figures 6B–F**, Wilcoxon rank sum test, *P* < 0.05). Compared to HC samples, *IFIT3*, *MX1*, *STAT1*, *STAT2*, and *OAS3* were all significantly upregulated in memory B cell clusters in SLE samples, and *IFIT3*, *MX1*, and *OAS3* were significantly upregulated in naïve CD4 T, TEM CD4 T, TEM CD8 T, MAIT T, and Mk clusters. In the CD14 mono, classical mono, and macrophage clusters, *IFIT3* and *STAT1* were notably upregulated. The PBMC scRNA-seq profiling of these biomarkers robustly supported that interferon-stimulated genes were likely to be the central pathogenesis of lupus patients.

We then compared the total PBMC transcript expression of each biomarker to assess disease activity (*PHACTR2*, *GOT2*, *SELL*, *CMC4*, *MAP2K1*, *CMPK2*, *ECPAS*, *SRA1*, and *STAT2*) between 16 SLE_A and 25 SLE_I samples. We found that the scRNA-seq results of *PHACTR2*, *SELL*, *CMPK2*, and *STAT2* for assessing SLE disease activity showed the same changes as the proteomics data in our study (**Figure S5B**, all *P* < 0.05). We then compared the differential expression of these four genes between SLE_A and SLE_I, as shown in violin plots (**Figures 6G–J**, Wilcoxon rank sum test, *P* < 0.05). Compared with HC samples, *PHACTR2*, *SELL*, *CMPK2*, and *STAT2* were all significantly upregulated in the CD14 mono-cluster in SLE_A samples. In Mk and TEM CD4 T cell clusters, *SELL*, *CMPK2*, *and STAT2* were significantly upregulated in SLE_A patients. These results indicate that molecular heterogeneity of SLE exists in different immune cells.

## Discussion

The ability to simultaneously screen a large number of proteins has changed the landscape of biomarker discovery research. In this study, we conducted PBMC proteomic profiling to identify specific alterations in SLE and identified two biomarker combinations that can classify SLE and assess disease exacerbation using the machine learning-based pipeline POC-SLE we have developed. Moreover, the accuracy of these biomarkers for SLE disease diagnosis and disease exacerbation assessment was further validated using ELISA. Finally, we obtained the immune cell subtypes of these biomarkers using PBMC scRNAseq. Therefore, these PBMC proteins can be further developed as clinical biomarkers, providing innovative tools for prompt clinical diagnosis and disease monitoring.

We found the interferon (IFN) signature, including IFIT3, MX1, STAT1, STAT2, and OAS3, as the main components of disease diagnosis biomarker combinations for SLE patients. In this study, we identified the IFN signature as a biomarker for disease diagnosis at the PBMC protein level, whereas most studies report the IFN signature at the transcriptional level as a measure of IFN activity (33). The central role of IFN signatures in SLE has been thoroughly investigated (34), and approximately half of SLE patients have an upregulated type I IFN gene signature (IGS) (35). The development of type I IGS as an SLE biomarker has been initiated. However, most studies have focused on the application of the IGS to help assess disease exacerbation, but not on SLE disease diagnosis. Baechler et al. found that an elevated IFN score is strongly associated with the most severe manifestations of SLE and that IGS is a marker for severe SLE (36). Feng et al. found that five IFN-inducible genes were highly expressed in SLE patients, and increased levels were correlated with SLE disease activity (37). Although most experiments show that IGS is associated with disease activity, none of them could demonstrate a connection between IGS and changes in SLEDAI-2K (38). Given that no significant differences in the disease activity index after anti-IFNα treatment were found (39), it is unreasonable to attribute the IFN signature in SLE as a biomarker reflecting disease activity. Furthermore, the expression levels of five IFN-inducible genes for SLE diagnosis were evaluated, and the modified IFN score may serve as a good biomarker for SLE diagnosis (40). The major concern of using the IFN signature for SLE diagnosis is specificity, because activation of the type I IFN pathway has been reported in other conditions, including rheumatoid arthritis, myositis, and primary Sjögren’s syndrome (41). In this study, we recruited patients with RA as disease controls to determine the specificity of the IFN signature for SLE diagnosis.

The IFN signature is known to be increased in PBMC of SLE patients (42), resulting in abnormal activation of different immune cells (43), and likely gives rise to an autoimmune response in SLE patients. Gao et al. found that OAS3 expression in CD4^+^ T cells was notably upregulated in active SLE patients compared to healthy participants (44), which was in keeping with our scRNA-seq results that OAS3 was increased in SLE naïve CD4^+^ T cells and TEM CD4^+^ T cells than in HC. IFIT3 is highly expressed in SLE of CD14^+^ monocytes and CD4^+^ T cells (45, 46), which is in line with the IFIT3 results from our scRNA-seq data. Furthermore, Li et al. identified that JAK-STAT pathway genes, including *JAK2*, *STAT1*, and *STAT2*, play vital roles in SLE pathogenesis (47). *STAT1* mRNA was increased in T cells (48), and total STAT1 protein was increased in B cells from SLE patients compared with healthy controls (49), which was in line with the *STAT1* result from our scRNA-seq data, including naïve B cells, memory B cells, and plasma cells. Furthermore, individual therapeutic agents can influence the expression of IFN signature-related biomarkers. Previous studies have shown that antimalarial-like drugs reduce interferon-stimulated gene expression in SLE PBMC *in vitro (* 50), including MX1. Furthermore, baricitinib treatment reduces the mRNA expression of functionally interconnected genes involved in SLE, including *STAT1*-target, *STAT2*-target, and multiple IFN-responsive genes (51). Hence, it has been suggested that the IFN signature plays an important role in SLE pathogenesis *via* various immune cells.

Of the ELISA-validated protein combinations for assessing disease exacerbation, including PHACTR2, GOT2, L-selectin, CMC4, MAP2K1, CMPK2, ECPAS, SRA1, and STAT2, the biomarker combination exhibited an ROC AUC value of 0.990 in terms of distinguishing active SLE patients from inactive SLE patients. Seven proteins (PHACTR2, GOT2, CMC4, MAP2K1, CMPK2, ECPAS, and SRA1) were reported for the first time as markers with a potential impact on SLE exacerbation. L-selectin (CD62L) is a member of the selectin family of adhesion molecules expressed in leukocytes (52). Soluble L-selectin (sL-selectin) is elevated in the serum (53) and cerebrospinal fluid (54) of patients in comparison to healthy donors. Levels of sL-selectin correlate significantly with the levels of antibodies to dsDNA in patients with SLE (55). Moreover, previous studies have shown that sL-selectin (55, 56) and L-selectin (57) levels are correlated with SLE disease exacerbation and have been suggested as useful biomarkers for assessing disease exacerbation. STAT2, a downstream signaling molecule of type I IFN, contributes to its transactivation domain for gene transcription (58). A study of single-cell gene expression in SLE monocytes revealed that the transcriptional expression level of STAT2 was most decreased in patients with a high SLEDAI (59). However, Ramírez-Vélez et al. found that differences in STAT2 phosphorylation between active and inactive SLE patients were not significant, and there was no correlation between SLE disease activity and STAT2 phosphorylation (60). Thus, the role of unphosphorylated and phosphorylated STAT2 in unleashing the IFN signature in SLE requires further investigation.

Our study has some limitations. First, although we used two different cohorts to generate consistent results, the inclusion of additional ethnic groups and a larger sample size would provide additional power to validate the PBMC proteins reported here. In addition, a longitudinal study should be designed to investigate how these PBMC biomarkers relate to treatment response over time and long-term outcomes. Finally, mechanistic studies are needed to elucidate their respective roles in disease pathogenesis.

## Conclusions

Our study found that a machine-learning pipeline can be used to identify biomarker combinations for disease diagnosis and assessment of disease exacerbation based on the PBMC proteome of patients with SLE. Furthermore, scRNA-seq analysis identified biomarkers from different immune cells, which can provide potential treatment targets for SLE patients.

## Data availability statement

The datasets presented in this study can be found in online repositories. The name of the repository and accession number can be found below: ProteomeXchange PRIDE Repository; PXD025076.

## Ethics statement

The studies involving human participants were reviewed and approved by the Institutional Review Board of ShenZhen People’s Hospital. The patients/participants provided their written informed consent to participate in this study.

## Author contributions

YD and LY designed and supervised the study. YL, SQ, SM, and WC collected the clinical samples and clinical measurement information. YL, WD, RC, XD, XH, and DL analyzed the clinical data. YL, BK, CY, and BH performed the mass spectrometry analyses. YL, CM, and SL conducted bioinformatics analyses and scRNAseq analyses. YL, DT, JH, LY and YD interpreted the data. YL wrote the manuscript. DT, JH, LY and YD edited the manuscript. All authors read and approved the final manuscript. All authors contributed to the article and approved the submitted version.

## Funding

This project was supported by the science and technology plan of Shenzhen (No. JCYJ20200109144218597), Shenzhen Key Medical Discipline Construction Fund (No. SZXK011), the Key Research and Development Program of Guangdong Province (No.2019B020229001), Sanming Project of Medicine in Shenzhen (No. SYJY201704 and No. SYJY201705), the National Natural Science Foundation of China (No.81971464) and Guangdong Science and Technology Projects (No. 2020A1313030112).

## Conflict of interest

Author LY was employed by Guangzhou Enttxs Medical Products Co., Ltd. Author BH was employed by Reproductive and Genetic Hospital of China International Trust and Investment Corporation (CITIC)-Xiangya.

The remaining authors declare that the research was conducted in the absence of any commercial or financial relationships that could be construed as a potential conflict of interest.

## Publisher’s note

All claims expressed in this article are solely those of the authors and do not necessarily represent those of their affiliated organizations, or those of the publisher, the editors and the reviewers. Any product that may be evaluated in this article, or claim that may be made by its manufacturer, is not guaranteed or endorsed by the publisher.
