# Supplementary material for: Combined proteomics and single cell RNA-sequencing analysis to identify biomarkers of disease diagnosis and disease exacerbation for systemic lupus erythematosus
Source: Front Immunol. 2022 Nov 29;13:969509. doi: 10.3389/fimmu.2022.969509 (PMC9746895; doi:10.3389/fimmu.2022.969509)
Supplement: Supplementary file 6 [file DataSheet_1.docx]

## Supplementary Figures


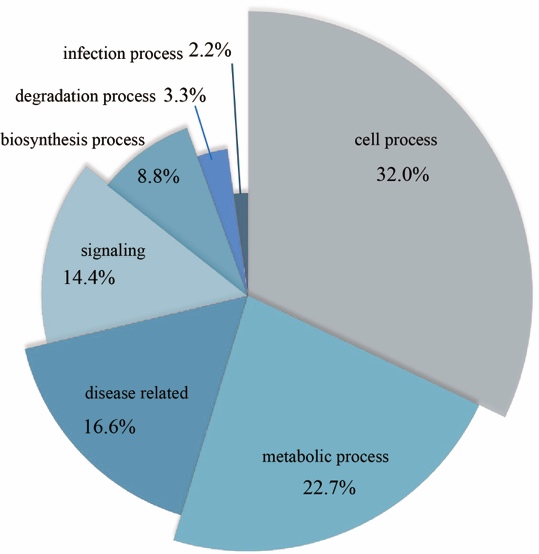


**Fig. S1. Classification of KEGG pathways in SLE patients by GSVA.**

**
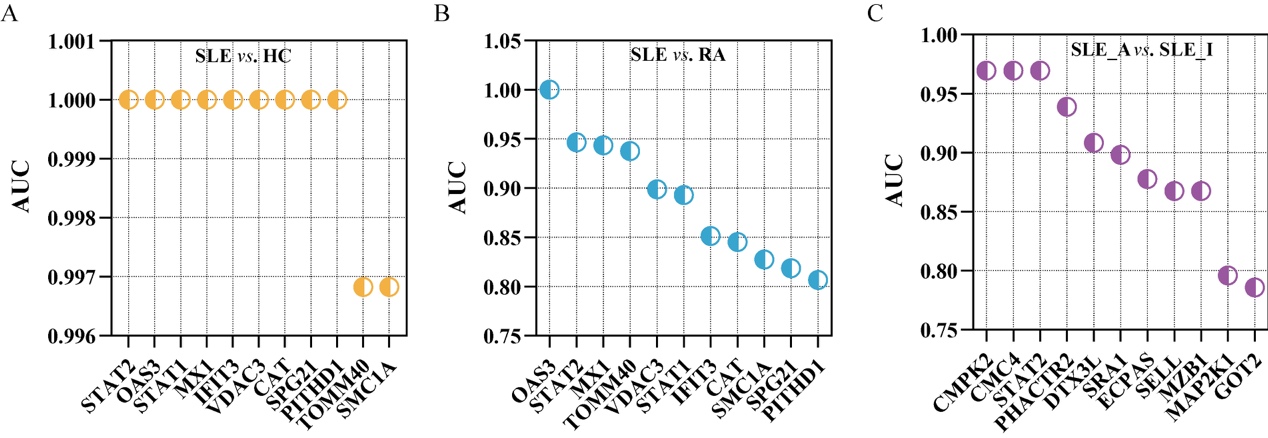
**

**Fig. S2. RFA AUC values of each candidate biomarkers.**

The RFA AUC values of each of the seven proteins to distinguish SLE from HC (A), SLE from RA (B). The RFA AUC values of each of the 11 proteins to distinguish SLE_A from SLE_I (C).

**
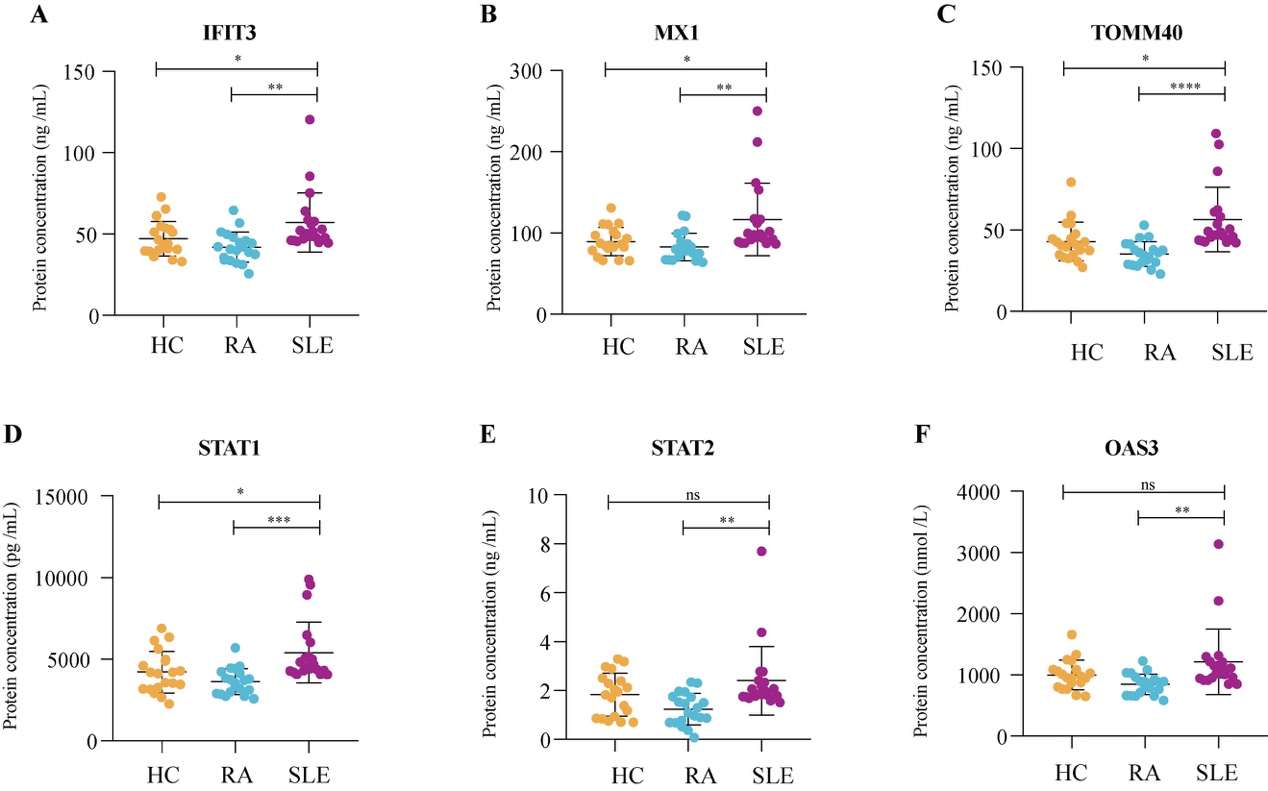
**

**Fig. S3** The PBMC protein concentration of biomarkers for SLE disease diagnosis measured by ELISA, including (A) IFIT3, (B) MX1, (C) TOMM40, (D) STAT1, (E) STAT2, and (F) OAS3. The statistics were calculated on biological replicates (20 samples for each HC, RA, and SLE group) with unpaired Student’s t-test (A-F). * *P* < 0.05; ** *P* < 0.01; *** *P* < 0.001; **** *P* < 0.0001; ns for non-significant.


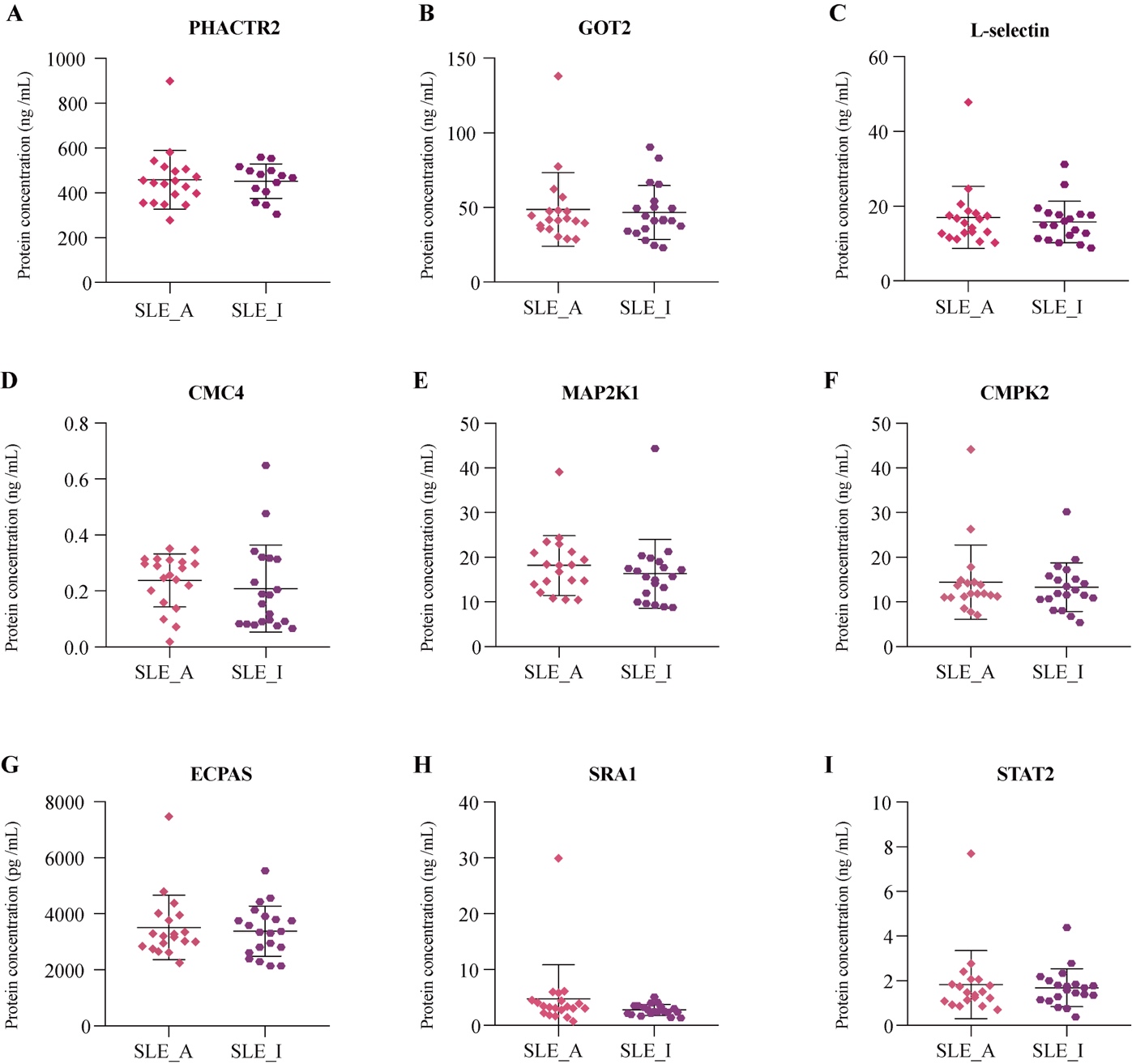


**Fig. S4** The PBMC protein concentration of biomarkers for assessing SLE disease exacerbation measured by ELISA, including (A) PHACTR2, (B) GOT2, (C) L-selectin, (D) CMC4, (E) MAP2K1, (F) CMPK2, (G) ECPAS, (H) SRA1, and (I) STAT2. The statistics were calculated on biological replicates with unpaired Student’s t-test (A-I). The comparision for nine protein concentration between SLE_A and SLE_I was all for non-significant.

**
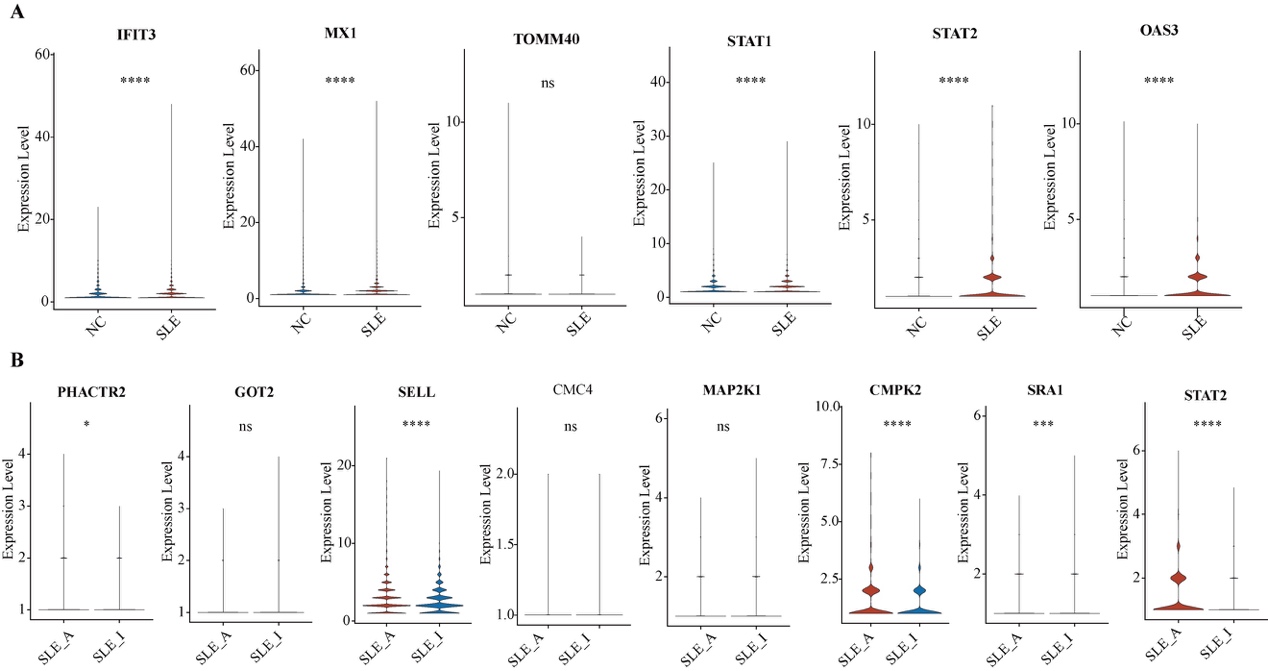
**

**Fig. S5 The transcriptome expression levels of SLE-related biomarkers in PBMC for SLE patients.**

(A) Violin plots showing the transcriptome expression level of SLE disease diagnosis related genes between SLE and HC; (B) Violin plots showing the transcriptome expression level of assessing disease exacerbation related genes identified between active SLE (SLE_A) and inactive SLE (SLE_I) patients. The statistics were calculated on biological replicates (21 samples for HC group and 46 samples for SLE group which included 16 active SLE and 25 inactive SLE) with Wilcoxon rank sum test. * *P* < 0.05; *** *P* < 0.001; **** *P* < 0.0001; ns, non-significant.
